# Cancer Stem Cells in Sarcomas: In Vitro Isolation and Role as Prognostic Markers: A Systematic Review

**DOI:** 10.3390/cancers15092449

**Published:** 2023-04-25

**Authors:** Maria Angeles Chico, Cristina Mesas, Kevin Doello, Francisco Quiñonero, Gloria Perazzoli, Raul Ortiz, Jose Prados, Consolacion Melguizo

**Affiliations:** 1 Institute of Biopathology and Regenerative Medicine (IBIMER), Center of Biomedical Research (CIBM), University of Granada, 18100 Granada, Spain; 2 Instituto Biosanitario de Granada (ibs. GRANADA), 18014 Granada, Spain; 3 Department of Anatomy and Embryology, Faculty of Medicine, University of Granada, 18071 Granada, Spain; 4 Medical Oncology Service, Hospital Universitario Virgen de las Nieves, 18014 Granada, Spain; 5 Department of Medicine, Faculty of Health Sciences, University of Almería, 04120 Granada, Spain

**Keywords:** sarcomas, cancer stem cells, drug resistance, biomarkers, systematic review

## Abstract

**Simple Summary:**

Cancer stem cells (CSCs) are responsible for the great challenge in the treatment of sarcomas due to their high prediction to form metastases. This systematic review focuses on collecting existing research on the expression of CSC markers in different types of sarcomas in both in vitro cell lines and patient samples. The results show a great heterogeneity of the studied markers, with ALDH being the only marker commonly used in sarcomas. This broader view may help to develop new CSC characterization assays to advance in more personalized treatments.

**Abstract:**

Sarcomas are a diverse group of neoplasms with an incidence rate of 15% of childhood cancers. They exhibit a high tendency to develop early metastases and are often resistant to available treatments, resulting in poor prognosis and survival. In this context, cancer stem cells (CSCs) have been implicated in recurrence, metastasis, and drug resistance, making the search for diagnostic and prognostic biomarkers of the disease crucial. The objective of this systematic review was to analyze the expression of CSC biomarkers both after isolation from in vitro cell lines and from the complete cell population of patient tumor samples. A total of 228 publications from January 2011 to June 2021 was retrieved from different databases, of which 35 articles were included for analysis. The studies demonstrated significant heterogeneity in both the markers detected and the CSC isolation techniques used. ALDH was identified as a common marker in various types of sarcomas. In conclusion, the identification of CSC markers in sarcomas may facilitate the development of personalized medicine and improve treatment outcomes.

## 1. Introduction

Sarcomas are a diverse group of neoplasms comprising over 100 subtypes, all originating from mesenchymal cells [1]. Although rare in adults (1%), sarcomas are becoming increasingly prevalent in children and adolescents, with an incidence of 15% of childhood cancers [2]. Currently, they are classified into three groups: (i) bone sarcomas (BS) (15%), which include chondrosarcoma, osteosarcoma, Ewing’s sarcoma, giant cell tumor, and others; (ii) soft tissue sarcomas (STS) (80%), with rhabdomyosarcoma, liposarcoma, leiomyosarcoma, synovial sarcoma, fibrosarcoma, desmoplastic small round cell tumor, and malignant peripheral nerve sheath tumor being the most frequent subtypes; and (iii) gastrointestinal stromal tumor (GIST) [1,3,4].

Despite current treatments (a combination of neoadjuvant and adjuvant chemotherapy with surgery), sarcoma prognosis and survival remain poor due to the high propensity of sarcomas to form metastases, even at the time of diagnosis, with undetectable micrometastases [5,6,7,8]. The high intratumoral heterogeneity contributes to the ineffectiveness of existing treatments [9]. Within the tumor, there is a subpopulation of cells exhibiting pluripotent embryonic stem cell characteristics, called cancer stem cells (CSCs), that are involved in tumor initiation, proliferation, recurrence, metastasis, and drug resistance [10,11,12,13]. This has sparked interest in developing an alternative approach to target the cells responsible for tumor and metastasis formation [14].

The use of CSCs as diagnostic and prognostic markers is increasingly important in various cancers. However, although some markers, such as Sox2, ALDH1, CD117, CD133, among others, have been described in sarcomas, there is no standardized differential pattern for each subtype that can be used clinically [15,16,17,18]. Additionally, more and more in vitro studies are being carried out with isolated CSCs to investigate their drug resistance and identify more effective treatments [19]. Hence, although numerous methodologies for the isolation of CSCs exist, there is a need to standardize the isolation methods and identify the most suitable CSC markers for each sarcoma subtype [11,20].

Given the importance of CSCs and their association with poor sarcoma prognosis, in addition to the great heterogeneity in the use of different markers in CSC research, this systematic review aims to collect all existing studies on the use of CSCs as prognostic biomarkers in sarcomas and the methods used to isolate and characterize these cells for preclinical in vitro studies in order to establish the markers that define CSCs in the different subtypes of sarcomas.

## 2. Materials and Methods

### 2.1. Study Eligibility

The objective of this systematic review was to collect the most recent and representative data on CSC markers in sarcoma cancer, as well as isolation techniques for this aggressive cell subgroup for in vitro culture. This review was performed in accordance to the PRISMA (Preferred Reporting Items for Systematic Reviews and Meta-Analyses) [21] guidelines and has not been registered. We collected the bibliography of the last 10 years and considered the literature from previous years as obsolete. More than half of the current bibliography on the subject was included according to the Burton–Kebler obsolescence index [22].

### 2.2. Inclusion Criteria

Research articles were included if they were published between January 2011 and June 2021, studied the expression of CSC markers in human sarcoma cell lines and/or human tumor tissue samples, and included the method of isolation and characterization of CSCs. Open access full-text research articles were also included. No language restriction was established.

### 2.3. Exclusion Criteria

Research articles were excluded if they were repeated among the different databases. Articles that did not study marker expression in sarcoma lines or that used non-human sarcoma cell lines or cells derived from xenografts or xenotransplantation were excluded. Non-original articles, including reviews, clinical cases, clinical trials, systematic reviews, conference proceedings, editorials, letters, notes, patents, and book chapters, were also excluded.

### 2.4. Data Sources

The present systematic review was carried out using the following databases: MedLars Online International Literature, through PubMed, SCOPUS, Web of Science, and Cochrane Library Plus. Medical Subject Headings (MeSH) were defined using the descriptive terms “sarcoma”, “cancer stem cells”, and “biological markers”. The final equation was (((“Sarcoma”[Mesh] OR “Sarcoma”[Title/Abstract] OR “Osteosarcoma”[Title/Abstract] OR “Rhabdomyosarcoma”[Title/Abstract] OR “chondrosarcoma”[Title/Abstract] OR “synovial sarcoma” [Title/Abstract] OR “epithelioid sarcoma”[Title/Abstract] OR “Gastrointestinal Stromal Tumors”[Title/Abstract] OR “Ewing’s sarcoma”[Title/Abstract] OR “uterine sarcoma”[Title/Abstract] OR “leiomyosarcoma”[Title/Abstract] OR “pleomorphic sarcoma”[Title/Abstract] OR “fibrosarcoma”[Title/Abstract] OR “angiosarcoma”[Title/Abstract] OR “liposarcoma”[Title/Abstract] OR “Myxofibrosarcoma”[Title/Abstract]) AND (cancer stem cells[MeSH Terms] OR cancer stem cell[MeSH Terms])) AND (biological markers[MeSH Terms] OR “biomarkers” [MeSH Terms]) AND (“2011/01/01”[PDAT]: “2021/05/28”[PDAT]))). The same strategy was followed in all the databases, adapting the equation when necessary. In addition, the bibliography of the selected articles was reviewed to include other works of interest that did not appear in the initial search.

### 2.5. Study Selection

**Figure 1:**
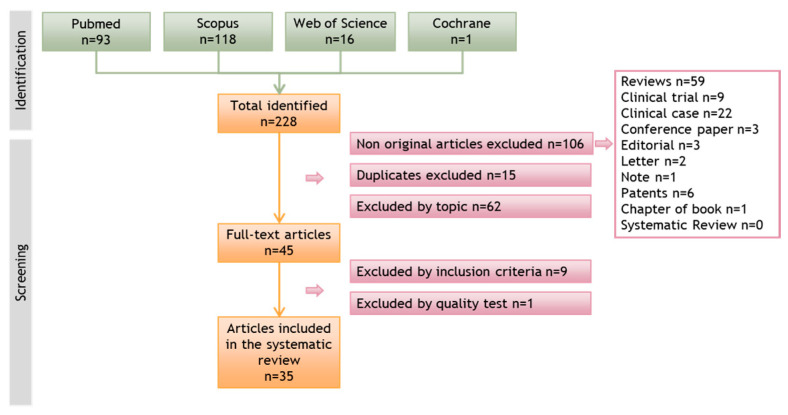
Flow diagram illustrating the search process and article screening in different databases that led to the selection of relevant articles for this systematic review.

Authors M.C. and C.M. conducted a bibliographic search in various databases and performed a first screening based on the titles and abstracts. The second step involved a complete reading of the research articles that passed the first screening. In both steps, the articles were evaluated based on the pre-established inclusion and exclusion criteria. In vitro studies were the focus of this review, and articles that only conducted in vivo studies were excluded at this stage. Figure 1 depicts the flow diagram of the selection process.

### 2.6. Data Extraction

Following the selection process, M.C. and C.M. independently extracted the data of interest from each research article. According to Cohen’s Kappa statistic test [23], there was good correlation between M.C. and C.M. Any disagreements were resolved through discussion until a consensus was reached. Otherwise, a third experienced author made the final decision. Each research article was subjected to a quality test for in vitro studies, which was divided into two phases. The first phase contained filters on the basic characteristics that an in vitro study should meet (score ≥ 5). Studies that did not meet this score were excluded. The second phase comprised questions on methodology, results, and conclusion. The studies were classified based on their score: low quality (score 0–5), medium quality (score 6–15), and high quality (score 16–20). Table 1 and Table 2 show the obtained data for ease of understanding. Both tables display the reference of the selected article, the type of sarcoma studied, the type of sample, the methodology of CSC extraction and characterization, and the main results.

## 3. Results

A total of 228 articles were identified in the initial search across the different databases consulted. After eliminating non-original articles (*n* = 106), duplicates between databases (*n* = 15), and articles that did not meet the inclusion criteria, 45 articles were retained for detailed analysis. Subsequently, 9 articles that did not meet the inclusion criteria and 1 that scored very low in the quality test were discarded. Finally, 35 articles were included in this systematic review. The flow diagram representing the search process and the screening of the articles is presented in Figure 1.

**Figure 2:**
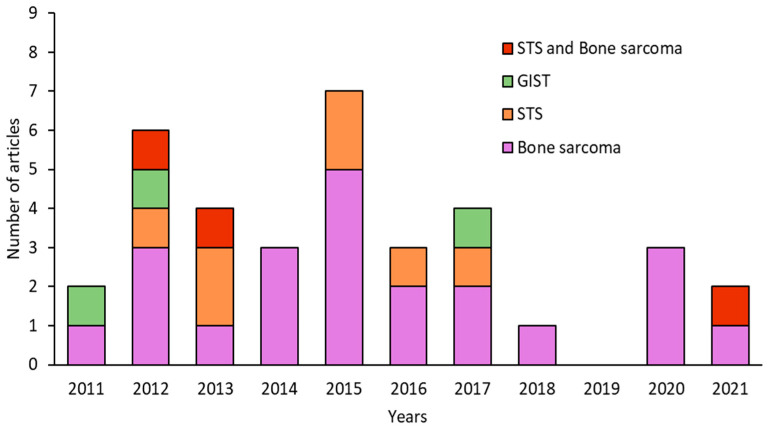
Number of articles published per year in the last decade on sarcomas, classified as BS, STS, or GIST.

**Figure 3:**
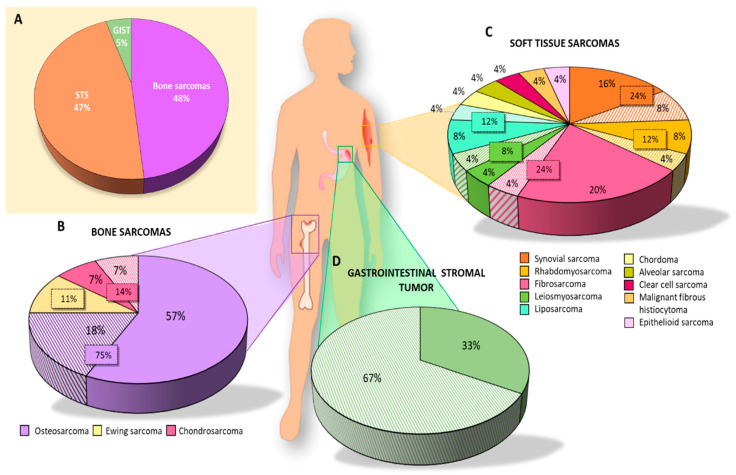
Graphic representation of the articles analyzed in this systematic review. Percentage of articles studying (**A**) each type of sarcoma; (**B**) each subtype of BS; (**C**) each subtype of STS; (**D**) GIST. Solid colors represent the results of articles in which CSCs were isolated using different techniques and the striped colors represent the results of articles on biomarker expression without isolation of CSCs.

The number of articles published on CSCs in sarcomas decreased over time. The years with the highest number of publications were 2012 and 2015 (6 and 7 per year, respectively), whereas the fewest publications were observed in 2018 (1 article) and 2019 (no articles) (Figure 2). Analysis of the type of sarcoma studied in the 35 articles published in the last 10 years revealed that 22 articles focused on BS, 7 on STS, and 3 on both types (i.e., STS and BS). GISTs have been less extensively studied with only 3 articles published in the last decade. Interestingly, more articles were published on BS than on STS, except in 2013 and 2014. However, articles on STS included a larger number of different subtypes, and the percentage of STS was similar to that of BS studied (Figure 3A). In the category of BS, most studies focused on osteosarcoma (75%), while on STS, synovial sarcoma (24%) and fibrosarcoma (24%) were the most studied subtypes, followed by rhabdomyosarcoma (12%) and liposarcoma (12%) (Figure 3B,C).

### 3.1. Different In Vitro Techniques for Isolating CSCs

#### 3.1.1. Bone Sarcoma

Of the 35 articles selected for this systematic review, 27 studied CSC markers for BS. Specifically, 21 isolated CSCs: 16 from osteosarcoma, 2 from chondrosarcoma, and 3 from Ewing’s sarcoma (Table 1). The percentage of the total studies collected on BS was 57%, 7%, and 11%, respectively (Figure 3B), with osteosarcoma being the most studied BS for isolating CSCs in vitro.

CSC markers in osteosarcoma were studied in 16 articles (Table 1). The most commonly used osteosarcoma cell lines were MG63, Saos-2, and U2OS, although most articles (10) utilized primary tumor samples from osteosarcoma patients to isolate CSCs. In addition, one article obtained samples from osteosarcoma lung metastasis [29]. Only four studies specified that patients had not been previously treated with chemotherapy [29,30,32,35], while patients underwent chemotherapy and radiotherapy in one study [46]. 

The most frequently used CSC isolation technique in osteosarcoma was Fluorescent-Activated Cell Sorter (FACS) combined with subsequent culture of the isolated and sorted cells in induction media for maintenance [25,27,31,37,46]. Four articles carried out the sphere formation assay using an induction culture medium [24,28,35,36], one article used FACS only without induction medium [33], two articles used the Magnetic-Activated Cell Sorting (MACS) technique and then induction medium [26,29], one article used MACS without induction medium [34], and finally, the Side Population (SP) technique with subsequent induction medium was used in three articles [13,30,32].

In the case of FACS and MACS techniques which were used in nine osteosarcoma articles, CSC markers were necessary for their isolation. The most commonly used method (three/nine articles) was double labeling with CD133 and CD44 [26,27,29], followed by labeling with CD133 only [34]. Other markers used were CD24 [25], CD49f [33], Sox2 together with Sca-1 [37] and CD109 [46]. Of the 13 osteosarcoma articles using induction and sphere-forming media, all utilized serum-free medium supplemented with factors, with EGF (epidermal growth factor) and FGF (fibroblast growth factor) being the most important, although in varying proportions. The most frequently used base medium was DMEM/F12, with a total of nine articles. One article used Ham’s F12 [24], while DMEM [27], RPMI [35], and N2B27 [37] were used depending on the cell line.

**Figure 4:**
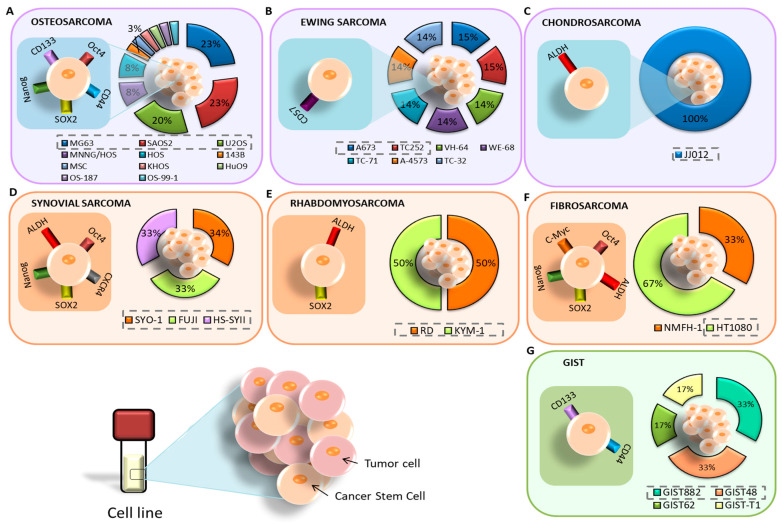
Cell lines and markers used for the study of CSCs in BS: (**A**) osteosarcoma; (**B**) Ewing’s sarcoma; (**C**) chondrosarcoma. Cell lines and markers used for the study of CSCs in STS: (**D**) synovial sarcoma; (**E**) rhabdomyosarcoma; (**F**) fibrosarcoma. (**G**) Cell lines and markers used for the study of CSCs in GIST.

All articles conducted characterization of CSCs after isolation. The most commonly used methods were RT-qPCR, Western blot, flow cytometry, IF, and ALDEFLUOR assay to check the expression of CSC markers in the isolated cells. In addition, in vivo tumorigenicity assays were performed. The most highly expressed markers of CSCs isolated from established cell lines were Oct4, CD133, SOX2, Nanog, and CD44 (Figure 4A). On the other hand, the most highly expressed markers in CSCs isolated from primary tumor samples from patients coincided with those mentioned for the cell lines. In the case of the only article that studied pulmonary metastasis, the CD133 and CD44 markers coincided in both the cell lines and patient samples [29]. Furthermore, several CSC markers have been associated with poor patient prognosis, including CD24 [25], DANCR [27], and CD49 [33]. Some articles also examined the modulation of certain markers after treatment. Zhou et al. [25] observed an increase in CD24 expression after treatment with Cisplatin, Epirubicin, and Hydrochloride. Epigallocatechin gallate (EGCG) reduced several CSC markers, namely CD44, CD133, SOX2, OCT4, c-Myc, and Nanog (CD133+/CD44+) [26]. Co-culture with mesenchymal stem cells (MSCs) also increased the expression of SOX2, OCT4, Nanog, and CXCR4 markers [28]. CSCs with high expression of CD248, CD133, OCT4, Nanog, and Nestin were more resistant to Doxorubicin, Cisplatin, and Methotrexate [30]. Verapamil treatment increased the sensitivity of CSCs to Doxorubicin [36].

Ewing’s sarcoma was the second most studied sarcoma subtype in terms of CSC isolation, with a total of three articles (11% of all BS) included in this review. There was no common cell line among these articles. Sphere formation using induction medium was the primary method of CSC isolation in two articles [38,39], while FACS isolation with subsequent culture in induction medium was applied in Emori et al. [46], using the CD109 marker. The medium composition was DMEM/F12 in two articles and IMDM in one [38]. The most common methods used for characterization were FACS, in vivo tumorigenicity, and RT-PCR, although SP and ALDEFLUOR assays were also applied. In Ewing’s sarcoma, CD57 was the most highly expressed marker in CSCs derived from patient samples [39], followed by OCT4 and Nanog [38] (Figure 4B). Additionally, the CD109 marker was expressed in both cell lines and patient samples [46].

Chondrosarcoma was the least studied BS, with a total of two articles (7% of all BS) included in this review. In both cases, FACS isolation was carried out using the ALDH marker [40,48], but only Granger et al. [40] subsequently cultured the isolated cells in induction medium. Common characterization methods included Western blot and RT-PCR, while IF was also applied in Lohberger et al. [48]. Both articles only used established cell lines. Granger et al. [40] found high expression of ALDH, CD44, STRO1, and STAT3 in the JJ012 line, while Lohberger et al. [48] found a high expression of ALDH, c-Myc, SOX2, B-Catenin, ABCG2, and ABCB1 in the SW1353 cell line (Figure 4C). Additionally, ALDH+ CSCs were found to be resistant to chemotherapy in Lohberger et al., 2012 [48], while treatment with PRP-1 (proline-rich polypeptide-1) had no effect in Granger et al. [40].

#### 3.1.2. Soft Tissue Sarcomas (STS)

Twenty-four of the 35 articles selected for this review studied CSC markers in STS. Of these, 16 articles isolated CSCs, 4 of which focused on synovial sarcoma (16% of all STS), 2 on rhabdomyosarcoma (8%), 4 on fibrosarcoma (16%), 1 on epithelioid sarcoma (4%), 1 on malignant fibrous histiocytoma, 1 on leiomyosarcoma (4%), 2 on liposarcoma (8%), and 1 on chordoma (4%). Synovial sarcoma and fibrosarcoma were the most studied STS in terms of CSC isolation.

**Figure 5:**
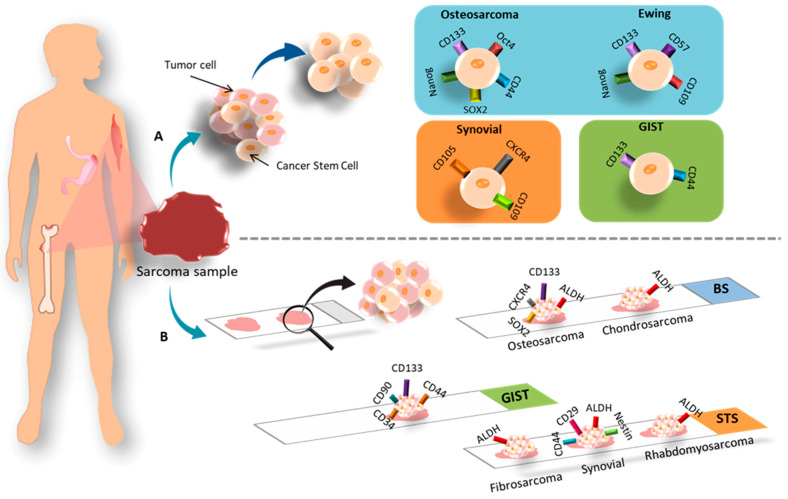
Representation of the most commonly used CSC markers in samples derived from patients with BS, STS and GIST: (**A**) CSC isolation; (**B**) histological techniques.

The isolation of CSCs from synovial sarcoma was described in four articles (Table 1). The most commonly used cell line was Fuji. FACS with induction media and the CD109 [46] and CD271 markers [47] were the most commonly used isolation methods, followed by FACS without induction media with the ALDH marker [48]. Sphere formation assay was also employed by Kimura et al. [41]. The induction medium used in all three articles was based on DMEM/F12. Characterization methods included RT-PCR, flow cytometry, Western blot, immunohistochemistry (IHC), and IF. From already established cell lines, Kimura et al. [41] found increased expression of the markers Nanog, Oct4, SOX2, and CXCR4 in CSCs from SYO-I, Fuji, and HS-SYII lines. Lohberger et al. [48] used the SW982 line, whose CSCs expressed ALDH, c-Myc, Sox2, B-catenin, and ABCG2 (Figure 4D). Emori et al. [46] did not find expression of CD119 in FUJI and YaFuss. One article showed expression of the unusual markers CD105, 2E4B4, 58B1, W5C4, and CD109 in both the SW982 line and in primary tumor samples from patients (Figure 5). In addition, the tumors expressed CD271 and TNAP [47]. Regarding chemotherapy treatment, SW982 ALDH+ cells were found to be resistant to Doxorubicin [48]. Furthermore, CD271+ cells derived from patient primary tumors and the SW982 line were found to be resistant to Doxorubicin and Epirubicin [47].

Fibrosarcoma, along with synovial sarcoma, was one of the most studied STS in the articles reviewed. Table 1 lists four articles on this topic. The most commonly used cell line was HT1080 [44,47] (Figure 4F). Three articles used FACS in different ways: with the ALDH marker and induction medium [43], with ALDH without subsequent culture in induction medium [48], or with the CD271 marker and subsequent induction medium [47]. MACS with the CD133 marker and subsequent induction medium was used in Feng et al., 2013 [44]. RPMI was used as induction medium in Li et al. [43], while the rest used DMEM/F12. The prevailing characterization methods were RT-PCR, Western blot, in vivo tumorigenesis, IF, and IHC. Only one article used primary tumor samples from patients in the study. Lohberger et al. [48] used the SW982 and SW684 lines, Wirths et al. [47] used SW982, SE872, and HT1080, Feng et al. [44] used HT1080, and Li et al. [43] used the NMFH-1 line. Three articles found a high expression of c-Myc and SOX2 markers in CSCs. Nanog, Oct4, and ABCG2 markers were found in two articles. Other markers expressed in cell lines include STAT3, CD133, BMi-1, ALDH, and B-catenin. Notably, BMi-1 was overexpressed in CD133+ CSCs in HT1080 [44], but not in ALDH+ NMFH-1 cells [43]. Both ALDH+ and CD133+ cells mentioned were found to be resistant to treatment with Doxorubicin and Cisplatin. Additionally, CD105, 2E4B4, 58B1, W5C4, and CD109 were expressed in cell lines and samples.

Isolation of CSCs from rhabdomyosarcoma and liposarcoma was carried out in two articles each once (Table 1). The studies used different cell lines: TE-671 [48], and RD and KYM-1 [42] (Figure 4E). None of the studies used patient samples, but both used FACS-based CSC isolation methods with the ALDH marker. The common characterization methods were RT-PCR, Western blot, IF, and in vivo tumorigenicity. One of the articles reported no results using the cellular line of rhabdomyosarcoma [48], and the other found overexpression of ALDHA3, ALDHB1, ALDHL2, SOX2, ABCG2, ABCB1, and ABCA2 markers. Additionally, ALDH+ cells were found to be more resistant to treatment with Vincristine, Cyclophosphamide, and Etoposide. Table 1 includes two articles that evaluated CSC isolation in liposarcoma [47,48]. Both studies used the SW872 cell line. Wirths et al. [47] isolated CSCs using FACS (CD271) and subsequently maintained the cells in induction medium. They characterized CSCs using IHC. On the other hand, Lohberger et al. [48] used FACS (ALDH) for isolation and IF Western blot and RT-PCR for characterization. The markers studied in both articles did not coincide. In Wirths et al. [47], both CSCs derived from the cell line and primary tumor samples from patients expressed CD105, 2E4B4, 58B1, W5C4, and CD109. The primary tumor tissue also expressed CD271 and TNAP, with CD271+ cells being associated with Doxorubicin resistance. On the other hand, Lohberger et al. [48] only isolated CSCs from the cell line, and these cells expressed ALDH, c-Myc, Sox2, B-catenin, and ABCG2 markers.

Finally, one article related to the isolation of CSCs from epithelioid sarcoma, malignant fibrous histiocytoma, leiomyosarcoma and chordoma was found. Table 1 presents a single article reporting the isolation of CSCs in epithelioid sarcoma [46]. The study employed two cell lines, FU-EPS-1 and VA-ES-BJ, in addition to primary tumor samples. Isolation of CSCs was carried out using FACS with the CD109 marker and subsequent culture in induction medium. CSCs were characterized using RT-PCR and the ALDEFLUOR assay. The CSCs obtained from the established cell lines and tumor samples expressed ALDH, SOX2, OCT4, Nanog, and CD109 markers. It is worth noting that the expression of CD109 correlated with low survival. On the other hand, the study by Emori et al. [46] used the malignant fibrous histiocytoma cell lines, MFH2033 and MFH2004, in addition to primary tumor samples. Isolation of CSCs was carried out using FACS with the CD109 marker and subsequent culture in induction medium. The CSCs were characterized using RT-PCR and the ALDEFLUOR assay. The CSCs obtained from the cell lines and tumor samples expressed CD109, which also correlated with poor survival. In relation to leiomyosarcoma, Table 1 includes the only article that investigated this tumor [47]. The study employed the SK-LMS1l cell line and tissue samples from the primary tumor. Isolation of CSCs was carried out using FACS with CD271, followed by induction media and characterization with IHC. The CSCs obtained from the cell lines and tumor samples expressed CD105, 2E4B4, 58B1, W5C4, and CD109 markers. The primary tumor tissue also expressed CD271 and TNAP, with CD271+ cells being associated with Doxorubicin resistance. Finally, Lohberger et al. [48] studied chordoma using the MUG-Chor-1 cell line and did not use patient samples. Isolation of CSCs was carried out using FACS with ALDH and subsequent characterization of CSCs with IF, Western blot, and RT-PCR. The isolated CSCs expressed ALDH, Sox2, cMyc, β-catenin, and ABCG2 markers.

#### 3.1.3. GIST

Only one of the three articles (33%) on GIST reported the isolation of CSCs (Figure 3D) [45]. The study employed four cell lines, namely, GIST882, GIST48, GIST62, and GIST-T1 (Figure 4G). Isolation of CSCs was performed by FACS using CD133 and CD44 markers, followed by characterization using IF, flow cytometry, and SP assay. The CSCs showed high expression of CD44 and CD133 markers, especially in GIST located in the stomach. The high expression of these markers was associated with resistance to Imatinib.

### 3.2. CSC Markers in Histological Samples

#### 3.2.1. Bone Sarcoma

This systematic review includes 27 articles on BS, of which 8 only measured CSC marker expression on the entire cell population without performing in vitro treatment on cell lines or patient tumor samples. Specifically, 18% of the articles focused on osteosarcoma, and 7% on chondrosarcoma. No articles were found on Ewing’s sarcoma. The results are summarized in Table 2.

**Figure 6:**
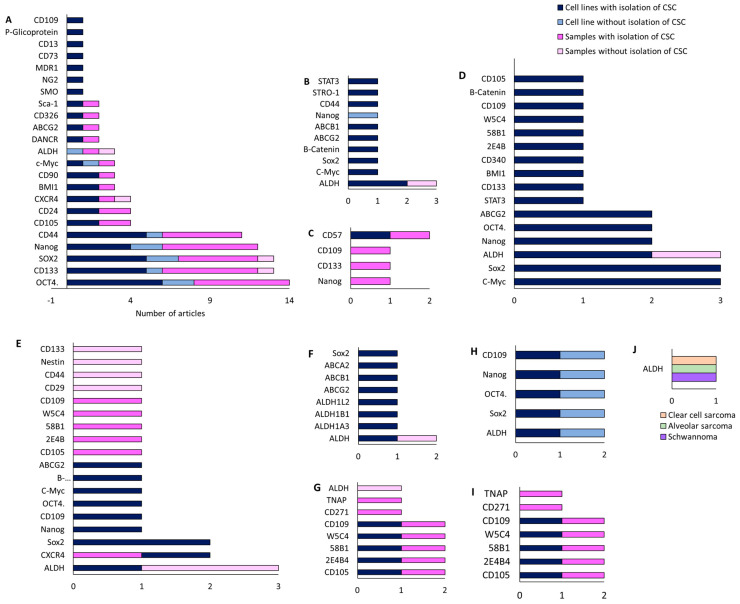
Number of articles that investigated CSC markers in cell lines after the CSC isolation process, in cell lines without CSC isolation, in patient samples after CSC isolation, and in patient samples without CSC isolation for different STS subtypes: (**A**) osteosarcoma; (**B**) chondrosarcoma; and (**C**) Ewing’s sarcoma; (**D**) fibrosarcoma; (**E**) synovial sarcoma; (**F**) rhabdomyosarcoma; (**G**) liposarcoma; (**H**) epithelioid sarcoma and malignant fibrous histiocytoma; (**I**) leiomyosarcoma; and (**J**) clear cell sarcoma, alveolar sarcoma, and schwannoma.

Analysis of CSC markers in osteosarcoma without isolation was found in four articles (Table 2). The commonly used cell lines were MG63 and U2OS, while two articles only used patient samples [49,57]. One article exclusively used patient samples [51]. The marker expression analysis was primarily based on Western blot, followed by RT-qPCR. One article performed FACS and ALDEFLUOR assay [57], and another article used IHC [49]. CD133 and CXR4 markers were expressed in 26% and 36% of the samples, respectively, and were found to be correlated with pulmonary metastasis [49]. In contrast, Avdonkina et al. [57] did not observe CD133 expression. Moreover, the modulation of markers by the SOX2lncRNA gene was studied; its overexpression increased OCT4, ALDH, CD133, and CD44 markers in Saos-2 cells, while the opposite was observed in U2OS cells. Overexpression of SOX2lncRNA was also associated with poor survival [50] (Figure 6A). Additionally, Zoledronate-resistant cells showed high expression of Nanog, c-Myc, Oct-4, and Sox2 markers [51].

Only one article studied CSC markers in chondrosarcoma [52] using the JJ012 cell line, without using patient samples. The authors analyzed Nanog expression using Western blot. Comparison of the baseline with the line after PRP-1 treatment revealed a significant decrease in the Nanog marker.

#### 3.2.2. Soft Tissue Sarcoma

Synovial sarcoma was studied in two articles focusing solely on patient samples and analyzing the markers using IHC [57] or FACS with CD133 and the ALDEFLUOR assay [53]. According to this study, CD133 was the most frequently expressed marker in 85% of the samples, followed by CD24 and CD44 in 55%, Nestin in 30%, and ALDH in 25%. ALDH was found to be correlated with poor survival. Interestingly, ALDH was expressed more frequently in STS than in BS [57]. On the other hand, only one article investigated undifferentiated cardiac sarcoma [54]. The study analyzed CD44 and OCT3/4 markers in patient samples using BS, and only CD44 expression was detected. Finally, we identified one article that studied several types of STS, including liposarcoma, fibrosarcoma, leiomyosarcoma, rhabdomyosarcoma, schwannoma, alveolar sarcoma, clear cell sarcoma, and dermatofibrosarcoma [57]. The study only analyzed patient samples using FACS and the ALDEFUOR assay. The results showed a higher expression of ALDH compared to BS, but no difference in CD133. 

#### 3.2.3. GIST

GIST was investigated in two articles. One study analyzed GIST882 and GIST48b cell lines, as well as patient samples [55], while the other only analyzed patient samples [56]. Immunohistochemistry was the main method used to investigate marker expression. The study by Geddert et al. [55] found that treatment with 5-aza-dC (5-aza-2’-deoxycytidine) reduced CD133 reactive hypomethylation and inversely correlated CD133 expression with survival, while directly correlating CD133 expression with gastric localization and KIT mutation. The relationship between KIT mutation and CD133 expression was also supported in the study by Bozzi et al. [56], as well as CD90, CD44, and CD34 markers. The expression of Kit, CD133, CD90, and CD34 was found to decrease or increase with the combination of KIT mutation and Imatinib treatment.

## 4. Discussion

The aim of this systematic review was to provide a detailed description of the existing literature on the expression of CSC markers in sarcomas, both in vitro and in the heterogeneous population of cells present in samples. To our knowledge, this is the first systematic review of CSC markers in sarcomas. After screening, 35 articles were selected. A great imbalance was found when comparing the types of sarcomas studied in each article. Specifically, 22 articles focused only on BS, while 7 focused on STS. Three articles studied both BS and STS, and the remaining studied GIST. These data do not agree with the classification of sarcomas since the largest group is composed of STS with 80%, followed by BS with 15% and GIST with 5% [3,4]. Therefore, although more research is needed in general in this field, specifically more studies are needed in STS. 

Focusing on STS, the most frequent subtype in children is rhabdomyosarcoma [58]. It is a very aggressive tumor with a skeletal muscle phenotype [59]. Two subtypes of rhabdomyosarcoma have been described: alveolar and embryonal, with the former being much more aggressive [60]. They present a 5-year survival rate of 28.9% and 74%, respectively [61]. On the other hand, the most frequent BS is osteosarcoma, which presents a peak incidence in adolescents, between 15 and 18 years of age, and another in adults at 42 years of age [62]. The 5-year survival rate in localized osteosarcoma is 65% and less than 20% in metastatic disease [63]. In this systematic review, only three articles on rhabdomyosarcoma have been found, so more studies are needed due to its high frequency. However, osteosarcoma is the most studied BS, which is consistent with its high prevalence.

This review specifically focuses on articles studying CSC markers in sarcomas. CSCs are defined as a subpopulation of cells in the tumor mass with the capacity for tumor initiation, progression, metastasis, and drug resistance [64,65]. Currently, the strategy followed by most studies is to first isolate CSCs and then analyze the expression levels of certain markers in these cells. CSCs can be isolated from existing cell lines or by generating cell cultures derived from patient tumor samples, which is not always an effective technique. In fact, the success rate in establishing cultures derived from patient samples is 82% in bone tumors and 68% in STS [66]. There are different techniques for the isolation of CSCs based on their unique characteristics, including drug resistance, dye explosion or SP assay, separation using surface markers with FACS or MACS, or ALDH activity. CSCs can also be enriched due to their ability to form tumor spheres in the absence of adherence and serum. However, there was no common method used in the studies reviewed, and even when the same method was used in sarcomas, there was much disparity in the methodology used. The CSC culture medium presented very different compositions, and no common marker was found to be used for isolation by FACS or MACS. In general, these processes select a population of CSCs with a specific characteristic, such as a particular marker, being separated from other subpopulations of CSCs with less expression of the same marker. Therefore, this CSC population is distanced from the heterogeneous population present in tumors in vivo. In conclusion, the studies were not carried out in the most appropriate way. Another strategy is based on analyzing CSC markers on the entire tumor population, either on in vitro cultures or on tumor samples using IF, IHC, or qPCR. This way, the entire CSC population present in the tumor can be analyzed. Of the 35 articles reviewed, only 9 used this approach, which is the most novel.

On the other hand, few studies analyzed the expression of CSC markers in tumor samples, so more studies are needed. It is necessary to compare the results of marker expression in isolated CSCs and in the whole cell population since the common markers could be the ones that should be used to corroborate that CSCs have been isolated in vitro similar to those present in the clinic. When analyzing the results of the included studies, the different homogeneity/heterogeneity between the markers detected in the different techniques used in the isolation of CSCs is very interesting. For example, in the case of GIST, CD44 and CD133 are constant in CSCs of this type of tumor, both in isolation using induction media and in biopsy studies. However, in the case of osteosarcoma and synovial sarcoma, a different marker profile can be seen in the case of biopsy studies compared to studies with induction media on cells. Thus, in the case of osteosarcoma, CD44 and Nanog are outstanding markers when isolation is carried out with induction media, while markers such as ALDH and CXCR5 are found on biopsy samples (Figure 6). Other markers such as CD133 and SOX2 are constant between studies. When we consider synovial sarcoma, CD44 is detected in biopsies but not in studies with induction media on the populations obtained. However, these results could be due either to a difference between the induced CSCs and those existing in the primary tumors, or it is possible that there is a bias related to the type of marker studied in each case, and the non-existence of results is due to the fact that such studies have not been carried out.

One of the most important findings from the reviewed studies is that ALDH is shown to be an important pan-CSC marker in the case of sarcomas, since it has been detected as positive in a multitude of sarcomas analyzed. Additionally, the use of chemotherapy seems to select some populations of CSCs with respect to others, with subpopulations of greater or lesser chemosensitivity and aggressiveness within the same CSCs analyzed, which could help to personalize therapies. However, there is a general lack of studies on which markers are more sensitive to different chemotherapeutic regimens. Only 30 articles in this review employed the use of chemotherapy treatment. This is important because it would enable us to determine which chemotherapy or radiotherapy to use according to the expression of each specific marker and to study resistance. 

Our results are based on studies of primary and metastatic tumor samples, with the aim of studying CSCs to achieve a treatment to prevent metastasis or otherwise prevent further spread of the disease. Other reviews in this field focus on more novel aspects such as the study of markers in circulating CSCs in blood, with the aim of developing a new therapeutic target for these circulating cells responsible for metastasis. These studies also highlight the significant problem that we are addressing and show that there is also great heterogeneity in the use of CSC markers in circulating cells [67,68,69,70,71,72]. Ultimately, the goal in the future is to achieve personalized medicine.

## 5. Conclusions

The role of CSCs in the prognosis of sarcomas has gained great importance in recent years. However, currently very few studies have investigated the expression of CSC markers in the diverse range of existing sarcoma subtypes. Therefore, further research is urgently needed to identify more sensitive CSC biomarkers and to establish a comprehensive set of markers that can accurately characterize CSCs in sarcomas. Ultimately, this will lead to the development of personalized treatments tailored to the specific CSC markers present in each patient’s tumor.

## Figures and Tables

**Table 1:** Isolation methodology and biomarkers expression of isolated CSCs from sarcoma cell lines and/or tumoral samples.

Type of Sarcoma (Ref.)	Sample	CSC Isolation Technique and Induction Medium	Methods for CSCs Characterization	Biomarkers	Treatment	Summary of Findings
Osteosarcoma [24]	Cell line: - Primary tumor: 1 patient	Spheres formation (Serum free 2× Ham’s F12 Coon’s medium supplemented with progesterone (20 nM), putrescine 100 µM, sodium selenite (30 nM), transferrin (25 µg/mL), insulin (20 µg/mL), EGF (10 ng/mL), b-FGF (10 ng/mL) were mixed with equal volume of 2% sterile methylcellulose).	- ALDH activity - Flow cytometric - RT-qPCR - IF	ALDH CD44 CD45 CD90 CD105 Nanog POU5F1 LIN28A SOX2 SATB2 KLF4 Nestin c-Kit PROM1 EZR AXL MYC	-	- CSC showed high levels of ALDH, CD44, CD105, CD90, Nanog, KLF4, SOX2, POU5F1, nestin, c-kit, and LIN28A compared to a fibroblast line primary cell line. - There was no expression of CD45 in CSC. - SATB2 and PROM1 showed expression in CSC and primary cell culture. - EZR, AXL, and MYC were expressed only in CSC. There was no expression in the primary cell lines.
Osteosarcoma [25]	Cell line: MG63 MNNG/HOS U2OS Primary tumor: 30 patients	- CSCs enriched with chemotherapy and then FACS (CD24). - Induction medium (Serum free DMEM/F12 supplement with 20 ng/mL EGF, 20 ng/mL FGF, and 20 ng/mL IGF.	- RT-qPCR - IF - Tumorigenicity in vivo	OCT4 NANOG SOX2 BMI1 CD24 CD14 CD117 CD133	Cisplatin Epirubicin Hydrochloride	- OCT4, NANOG, SOX2, BMI1, and CD24 expression in the spheres were higher than parental cells, but did not increase CD117 expression. - After the chemotherapy treatment, the proportion of CD24+ cells increased. CD117 expression was increased in MNNG/HOS, U-2 OS, and OSC228 cell lines, but low in MG63. - CD24 expression was higher in clinical osteosarcoma samples than in non-tumor tissues. CD24 is correlated with poor prognosis. - OCT4, NANOG, SOX2, and BMI1 expression in CD24+ cells rather than CD24- clinical samples.
Osteosarcoma [26]	Cell line: SaoS2 U2OS Primary tumor: -	- MACS (CD133+/CD44+) - Induction medium (serum-free DMEM-F12 medium supplemented with 20 ng/mL FGF, 20 ng/mL EGF, B27 (1:50), N2 (1:100), and 10 ng/mL LIF.	- qRT-PCR - Western blot - Tumorigenicity in vivo	SOX-2 OCT-4 c-Myc Nanog CD44 CD133	EGCG	- CD44, CD133, SOX2, OCT-4, c-Myc, and Nanog expression were significantly higher in CSC (CD133+/CD44+) than in the parental cells. - EGCG reduces the expression of these CSC markers.
Osteosarcoma [27]	Cell line: MG63 U2OS SaOS2 HOS 143B Primary tumor: 34 patients	- FACS (CD133+/CD44+) - Induction medium (serum-free DMEM medium supplemented with20 μg/L EGF, 20 μg/L FGF, 4U/L insulin, and 100 U/mL penicillin/streptomycin.	- qRT-PCR - -	CD133 CD44 SOX2 CD90	DANCR gene	- DANCR expression positively correlates with CD133, CD44, SOX2, and CD49 in sarcoma tissue and cell lines. - CD133+ and CD44+ cells showed a higher level of DANCR expression. - The expression of DANCR is related to poor patient survival.
Osteosarcoma [28]	Cell line: HOS MG63 MSC	Spheres formation with serum-free DMEM-F12 medium with progesterone (20 nM), putrescine (10 mg/mL), sodium selenite (30 nM), apo-transferrin (100 mg/mL), and insulin (25 mg/mL), EGF (20 ng/mL), and FGF (10 ng/mL).	RT-PCR	Nanog Oct4 SOX2 CXCR4	Co-culture with MSC	- CSC spheres showed higher expression of Sox2, Oct4, Nanog, and CXCR4 than the parental cells. - Expression is increased if csc is grown with MSC.
Osteosarcoma [29]	Cell line: MG-63 Saos-2 U2OS Primary tumor: 15 samples of patients with sarcoma lung metastasis	- MACS (C133/CD44) - Induction medium (Serum free DMEM supplemented with 1% methylcellulose, progesterone (10 nM), putrescine (50 mM), sodium selenite (15 nM), transferrin (13 mg/mL), insulin (10 mg/mL) and EGF (10 ng/mL), and FGF (10 ng/mL).	- RT-PCR - Western blot - Flow cytometry	CD133 CD44 Oct4 NANOG CXCR4	-	- CD133 and CD44 were expressed in the cell lines and lung metastases. - Increased expression of Oct4, Nanog, and CXCR4 was found in CD133+ and CD44+ cells. - CD133+ and CD44+ cells have a greater capacity for sphere formation, invasion, migration, and metastasis.
Osteosarcoma [30]	Cell line: - Primary tumor: 10 patients	- SP - Induction medium (Serum free 2 × DMEM/F12 with progesterone (20 nM), putrescine (100 μM), sodium selenite (30 nM), transferrin (25 μg/mL), insulin (20 μg/mL), and EGF (10 ng/mL) and bFGF (10 ng/mL)).	- IF - RT-qPCR - Western blot	CD248 CD133 Oct3/4A Nestin Nanog ABCG2 ABCB2 ABCA1 ABCB1	Doxorubicin Cisplatin Methotrexate	- SP cells showed higher expression of CD248, CD133, Oct3/4A, Nanog, and Nestin than non-SP cells of the tumor tissues. - SP cell spheres expressed CD133 and Oct-3/4A. - SP cells were more resistant to chemotherapy than non-SP cells. - SP cells showed higher expression of ABC transporter than non-SP cells.
Osteosarcoma [31]	Cell line: Saos-2 U-2 OS MG-63 Primary tumor: -	- FACS (CD133+) - Induction medium (serum-free DMEM-F12 supplemented with 20 ng/mL FGF, 20 ng/mL EGF, 1 × B27, 1 × ITS).	- RT-qPCR - Tumorigenicity in vivo	CD133 BMI-1 c-Myc Oct-4 SMO NG2	AP-SAL-NP	- CD133+ cells showed that the level of expression of CD133, Oct4, SMO, NG2, and BMI-1 were higher than CD133- and parental cultures. - AP-SAL-NP reduced the number of colonies and showed cytotoxicity toward CD133+ cells.
Osteosarcoma [32]	Cell line: - Primary tumor: 10 patients	- SP - Induction medium (Serum-free DMEM/F12 medium supplemented with N2, EGF (10 ng/mL) and hFGF (10 ng/mL)).	- Western blot - IF - RT-qPCR	CD133 Oct-4 Sox2 Nanog Nestin ABCG2	-	- Side population cells of CSC exhibited enhanced expression of ABCG2 compared with the non-side population. - CD133, Oct4, Sox2, Nanog, Nestin, and ABCG2 are overexpressed in side population cells.
Osteosarcoma [33]	Cell line: KHOS Primary tumor: 4 patients	FACS (CD49f+)	- Flow cytometry - Western blot - Tumorigenicity in vivo	CD44 CD90 CD49b CD105 CD117 CD49f	Doxorubicin Cisplatin	- Cell line cultures and patients showed high expression of CD44, CD90, and CD105, and low expression of CD117 and CD49f. - CD49f possible biomarker.
Osteosarcoma [34]	Cell line: Saos-2 Primary tumor: 55 patients: 4 parosteal 13 parosteal 12 chondroblastic 26 osteoblastic	MACS (CD133+)	RT-PCR	CD133 SOX2 MDR1	-	- Fibroblastic, parosteal, chondroblastic, and osteoblastic osteosarcoma samples and the cell line showed CD133+ cells. - CD133+ cells showed higher expression of Sox2 and MDR1 than CD133- cells from Saos-2.
Osteosarcoma [35]	Cell line: Saos-2 HuO9 Primary tumor: 1 patient (CHA59)	Spheres formation (serum free RPMI-1640 containing 15% KnockOut Serum Replacement and 2 mM L-glutamine).	- RT-PCR - Flow cytometry - ALDH activity - Tumorigenicity in vivo	PPARG ETS1 WNT1 WNT5B SOX2 NANOG POU55F1 Nestin ALDH CD24 CD44 CD133 CD166 ABCB1 ABCC1 ABCG2	Cisplatin 5-fluorouracil Imatinib	- The spheres showed higher clonogenicity and tumorigenicity than adherent cultures. - CD326, CD24, CD44 showed lower expression in adherent cells than in CHA59 and Saos-2 spheres. - ABCG2 and CBX3 showed higher expression in adherent cells than in CHA59 and Saos-2 spheres.
Osteosarcoma [36]	Cell line: MNNG/HOS SAR-OS (culture of adherent sphere-derived cells) Primary tumor: -	Spheres formation (serum-free DMEM/F12 medium supplemented with 1% of methylcellulose supplemented with 1% penicillin/streptomycin, 20 nM progesterone, 100 μM putrescine, 1% ITS, 10 ng/mL FGF, and 10 ng/mL EGF).	- Flow cytometry - Western blot - Tumorigenicity in vivo	CD105 CD73 CD13 CD90 CD34 CD44 CD11b CD19 HLA-DR Oct4 Nanog P-Glycoprotein BCRP	Doxorubicin Cisplatin Methotrexate Verapamil	- Spheres showed expression of CD73, CD90, CD13, and CD105. There was no expression of CD34, CD44, CD11b, CD19, and HLA-DR in SAR-OS. - Oct4, Nanog, P-Glycoprotein, and BCRP expression were higher in the spheres in comparison with the adherent cells in HNNG/HOS. - Spheres were more resistant to Doxorubicin, Cisplatin, and Methotrexate than adherent cells. Drug sensitivity was similar in SAR-OS and adherent parental cells. - Pretreatment with verapamil increased doxorubicin sensitivity of the spheres, whereas it did not affect the adherent cells.
Osteosarcoma [37]	Cell line: U-2OS MG63 HOS OS-187 OS-99-1 Saos-2 Saos-2-LM7 Primary tumor: 18 patients	- FACS (Sox2/Sca-1) - Induction medium (N2B27 defined serum free medium).	- Flow cytometry - RT-qPCR - Western blot - Tumorigenicity in vivo	Sox2 Sca-1	-	- All lines showed SOX2 overexpression compared to osteoblasts. - All tumor lines showed high Sox2 expression. - Sox2 deletion in the Saos-2-LM7 cell line and in lines derived from tumor tissue samples reduced Sca-1 expression and sphere formation.
Osteosarcoma [13]	Cell line: - Primary tumor: 6 Primary tumor	- SP - Induction medium (serum free 2 × DMEM/F12 supplemented with progesterone (20 nM), putrescine (100 μM), sodium selenite (30 nM), transferrin (25 μg/mL), insulin (20 μg/mL), EGF (10 ng/mL), and FGF (10 ng/mL) were mixed with an equal volume of 2% methylcellulose).	- RT-PCR - Tumorigenicity in vivo	ABCA2 ABCB1/MDR1 ABCC1/MRP1 ABCG2 Oct-4 Nanog CD44 CD117 CD133	Doxorubicin Cisplatin Methotrexate	- Sp cells formed spheres, but the non-sp cells did not form spheres. - Sp cells were more resistant to Doxorubicin, Cisplatin, and Methotrexate than non-SP cells. - ABCA2,ABCB1/MDR1, ABCC1/MRP1, ABCG2, Oct4, and Nanog expression were higher in SP cells in comparison with non-SP cells. - Sp and non-sp cells showed a similar expression of CD133, CD117, and CD44.
Ewing’s sarcoma [38]	Cell line: A673 TC252 Primary tumor: 4 patients	Spheres formation (IMDM supplemented with 20% KO serum, 10 mg/mL LIF, 10 ng/mL recombinant human EGF, and 10 ng/mL recombinant human FGF)	- RT-PCR - FACS - Tumorigenicity in vivo	CD133 Nanog Oct4	Doxorubicin Enoxacin	- Doxorubicin inhibited the growth of the adherent cells but not of the spheres derived from the tumors. - Enoxacin inhibited the growth of the spheres but not of the adherents cells derived from the tumors. Enoxacin induced CD133+ cell death. - The expression of Nanog and OCT4 was higher in the spheres than adherent cells of the 4 tumors. - Doxorubicin/enoxacin combination therapy showed synergy in targeting different cell populations.
Ewing’s sarcoma [39]	Cell line: VH-64 WE-68 TC-71 A-4573 TC-32 Primary tumor: 4 patients	Spheres formation (serum-free DMEM/F12 (1:1) supplemented with 4% B27, 20 ng/mL rhEGF, 20 ng/mL LIF, and 10 IE/mL (5 μg/mL) heparin).	- Flow cytometry - SP - Tumorigenicity in vivo	CD99 CD117 CD133 CD57	_	- There was no difference in CD99, CD117, CD133, and CD57 expression between spheres and adherent cells. - In cultures derived from tumor tissues, the expression of CD57 in spheres was higher than adherent cells.
Chondrosarcoma [40]	Cell line: JJ012 Primary tumor: -	- FACS (ALDH) - Induction medium (serum-free DMEM/F12 medium supplemented with 10 ng/mL FGF, 10 ng/mL EGF, and 10 µL/mL N2).	- qRT-PCR - Western blot	ALDH CD44 STRO-1 STAT3	PRP-1	- PRP-1 inhibited proliferation of CSCs. - ALDH+ cells showed lower growth in the presence of increasing doses of PRP-1 than ALDH- cells. - CD44, STRO-1, and STAT3 were expressed in CSCs. - No differences were found in CSC biomarkers expression between PRP-1-treated and untreated groups.
Synovial sarcoma [41]	Cell line: SYO-1 Fuji HS-SYII Primary tumor: 39 patients	Spheres formation (Serum free Medium/F12 supplement with 10 ng/mL FGF and 20 ng/mL EGF).	- RT-pPCR - Flow cytometry	NANOG OCT4 SOX2 CXCR4	-	- There was a higher expression of NANOG, OCT4, SOX2, and CXCR4 markers in spheres than in adherent cells of the cell lines. - The higher expression of CXCR4 was due to the induction of the spheres, not to pre-existing positive cells. - CXCR4-positive cells tended to form a cluster in the tumor mass. It correlated with a poor survival. - CXCR4 is a biomarker of synovial sarcoma.
Rhabdomyosarcoma [42]	Cell line: RD KYM-1 Primary tumor: -	FACS (ALDH)	- RT-qPCR - Tumorigenicity in vivo	ALDH1 ALDH1A1 ALDH1A2 ALDH1A3 ALDH1B1 ALDH1L1 ALDH1L2 c-Myc Sox2 ABCG2/BCRP ABCB1/MDR1 ABCA2	Vincristine Cyclophosphamide Etoposide	- The viability of cultures with Vincristine, cyclophosphamide, and etoposide is higher in the ALDH1^+^ cells than the ALDH1^−^ cells. - The expression of ALDH1A3, ALDH1B1, ALDH1L2, Sox2, ABCG2/BCRP, ABCB1/MDR1, and ABCA2 in the ALDH1^+^ cells increased compared to ALDH1^−^ cells. There was no difference in ALDH1A1 and c-myc expression. - The sample after chemotherapy exhibited a greater ALDH1 expression than before chemotherapy.
Fibrosarcoma [43]	Cell line: NMFH-1 Primary tumor: -	- ALDH activity - Induction medium (serum-free RPMI-1640, each well contained 20 μg/l of EGF and FGF).	- qPCR - Western blot - Tumorigenicity in vivo	ALDH1 c-Myc Bmi-1 Sox2 Nanog OCT3/4 STAT3 ABCG2	Doxorubicin Cisplatin	- The ALDH+ cells had more expression of ALDH1, c-Myc, STAT3, Sox2, Nanog, Oct3/4, and ABCG2 than ALDH- cells. - ALDH+ cells show improved sphere formation ability. - ALDH+ cells were more resistant to Doxorubicin and cisplatin in comparison with ALDH- cells. - ALDH+ cells show improved sphere formation ability. The spheres of ALDH+ cells were more resistant to Doxorubicin and Cisplatin than the spheres of ALDH- cells. - There was no difference in Bmi-1 expression between the ALDH+ and ALDH− cells.
Fibrosarcoma [44]	Cell line: HT1080 Primary tumor: -	- MACS (CD133+) - Induction medium (serum-free 2 × DMEM/F12 supplemented with progesterone (20 nM), putrescine (100 μM), sodium selenite (30 nM), transferrin (25 μg/mL), insulin (20 μg/mL), EGF (10 ng/mL), and FGF (10 ng/mL), mixed with an equal volume of 2% methylcellulose).	- RT-PCR - Western blot - Tumorigenicity in vivo	CD133 Nanog Oct3/4 SOX2 ABCG2 c-Myc Bmi-1	Cisplatin Doxorubicin	- CD133+ cells formed spheres while CD133- cells did not proliferate. - CD133+ cells were more resistant to doxorubicin and cisplatin than CD133- cells. In addition, spheres were more resistant than adherent cells. - CD133+ cells showed higher expression of Nanog, Sox2, Oct3/4, c-Myc, Bmi-1, and ABCG2 compared to CD133- cells.
GIST [45]	Cell line: GIST882 GIST48 GIST62 GIST-T1 Primary tumor: 131 GIST primary tumor 25 soft tissue sarcoma (microarrays)	FACS (CD133+/CD44+)	- IF - Flow cytometry - SP	CD133 CD44	Imatinib	- A higher expression of CD133 and CD44 was found in GIST compared to soft tissue sarcoma. Both markers were more expressed in tumors located in the stomach. Tumors sensitive to imatinib showed a lower expression of CD133. - Cell lines sensitive to imatinib showed lower expression of CD133 and CD44 than resistant lines (GIST48 and GIST62).
Osteosarcoma Ewing’s sarcoma Synovial sarcoma Epithelioid sarcoma Malignant fibrous histiocytoma [46]	Cell line: NY U2OS HOS OS2000 KIKU SKES WES RDES FUJI YaFuSS FU-EPS-1 VA-ES-BJ MFH2003 MFH2004 Primary tumor: 81 patients	- FACS (CD109) - Induction medium (DMEM/F12 medium with 10 ng/mL EGF, 10 ng/mL FGF, and 2% B-27).	- RT-PCR - ALDH activity	ALDH1 CD109 Sox2 Oct3/4 Nanog Twist 1 Snail 1	-	- Epithelioid sarcoma cell lines showed a higher proportion of ALDH1+ cells than the rest. The culture derived from the ESX showed an even higher proportion. - ALDH1- cells from ESX showed higher expression of Sox2, Oct3/4, and Nanog than ALDH1+ cells. In addition, they also showed higher expression of Twist 1 and Snail 1. - CD109 expressed in FU-EPS-1, VA-ES-BJ, OS2000, KIKU, NY, U2OS, SKES, WES, MFH2003, and MFH2004 cell lines. - CD109+ ESX cells showed higher sphere-forming capacity than CD109- cells. Poor survival correlated with higher CD109 expression.
Fibrosarcoma Leiomyosarcoma Liposarcoma Synovial sarcoma [47]	Cell line: HT1080 SK-LMS1l SW872 SW982 Primary tumor: 12 patients: LMS (*n* = 4), rhabdomyosarcoma (*n* = 4), and liposarcoma (*n* = 4)	- FACS (CD271) - Induction medium (DMEM, EMEM, or DMEM/F-12 with 1% penicillin/streptomycin, 2% B27 supplement, 0.1% EGF, 0.1% FGF, and 0.014% heparin).	IHC	CD105 2E4B4 58B1 W5C4 CD109 CD340 CD164 CD56 (W1C3) W4A5 W7C6 CD271 CD140b CD56 NPC CD10 CD318 CD344 F9-3C2F1 HEK3D6 CD172a CD349 W3D5A9 W5C5 TNAP CD117 CD133 CD326 CD34 CD324 W3C3	Doxorubicin	- CD105, 2E4B4, 58B1, W5C4, and CD109 expression was shown in all cell lines and tumor tissue. - CD340, CD164, W1C3, W4A5, W7C6, and CD271 were expressed in subpopulations of all cell lines and tumor tissue. - Differential expression of CD140b, CD56, NPC, CD10, CD318, CD344, F9-3C2F1, HEK3D6, CD172a, CD349, W3D5A9, W5C5, and TNAP was found. - No expression of CD117 and CD133, as well as CD326, CD34, CD324, and W3C3, was found in any cell line and tumor tissue. - Immunohistochemistry showed expression of CD271 and TNAP in leiomyosarcoma, rhabdomyosarcoma, and liposarcoma tumor samples. - CD271+ cells show higher proliferative activity, sphere-forming capacity, and higher resistance to Doxorubicin than CD271- cells in cell lines and tumor samples.
Fibrosarcoma Liposarcoma Synovial sarcoma Chondrosarcoma Rhabdomyosarcoma Chordoma [48]	Cell line: SW-684 SW-872 SW-982 SW-1353 TE-671 MUG-Chor1 Primary tumor: -	FACS (ALDH)	- IF - Western blot - RT-PCR	ALDH1 c-Myc β-catenin SOX2 ABCG2/BCRP1 ABCA2 ABCB1/MDR1	Doxorubicin Epirubicin Cisplatin	- There was a higher proportion of ALDH1-positive cells in the SW-684 cell line, SW-982 cells, and SW-1353 cells. - ALDH1+ cells showed higher expression of c-Myc, Sox2, and β-catenin than ALDH1- cells. - ABCG2 transporter showed higher expression in ALDH+ cells in all lines. ABCB1 only showed higher expression in the SW-1353 line. ABCA2 was not significantly expressed. - ALDH1+ cells of SW-1353 and SW-982 lines showed higher resistance to doxorubicin and epirubicin than ALDH1- cells. There were no significant differences in treatment with cisplatin.

Fibroblast growth factor (FGF), epidermal growth factor (EGF), magnetic-activated cell sorting (MACS), fluorescent-activated cell sorter (FACS), proline rich polypeptide-1 (PRP-1), epigallocatechin gallate (EGCG), mesenchymal stem cells (MSC), insulin-transferrin-selenium (ITS), AP-SAL-NP (PLGA nanoparticles loaded with salinomycin and CD133), recombinant human epidermal growth factor (rhEGF), leukemia inhibitory factor (LIF), epithelioid sarcoma tumor sample (ESX), immunofluorescence (IF), side population (SP), insulin-like growth factor (IGF), gastrointestinal stromal tumors (GIST), soft tissue sarcoma (STS).

**Table 2:** Biomarkers expression without isolation of sarcoma cell lines and/or tumor tissue.

Type of Sarcoma (Ref.)	Sample	Biomarkers	Technique	Treatment	Summary of Findings
Osteosarcoma [49]	Cell line: - Primary tumor 50 patients	CXCR4 CD133	IHC	-	- CD133 and CXCR4 were expressed in the plasma membrane and cytoplasm of tumor cells. - High CD133 expression was detected in 26% of the tumors. - Only 36% showed CXCR4 expression. - CD133 and CXCR4 expression (20.78% of the tumor) was correlated with lung metastasis.
Osteosarcoma [50]	Cell line: Saos-2 MG-63 U-2 OS MNNG/HOS Primary tumor: 138 patients	SOX2 OCT4 NANOG ALDH1 CD44 CD133 lncRNA SOX2-OT	- RT-pPCR - Western blot	-	- lncRNA SOX2-OT was overexpressed in tumor samples and cell lines compared to healthy tissues and non-tumor cells. - High expression of SOX2-OT LncRNA was associated with poor survival. - Low expression of SOX2-OT LncRNA inhibited Sox2 marker in U-2 OS, while high expression of SOX2-OT LncRNA elevated SOX2 expression. - The high expression of SOX2-OT LncRNA increased the expression of OCT4, ALDH1, NANOG, CD133, and CD44 in Saos-2 cells. Inhibition of LncRNA SOX2-OT expression decreased biomarker expression in U-2 OS.
Osteosarcoma [51]	Cell line: MG63 Primary tumor -	NANOG c-MYC OCT-4 SOX2	- Western blot	Zoledronate	- Zoledronate-resistant MG63 showed higher expression of Nanog, c-Myc, Oct-4, and Sox2 than the parental cells.
Chondrosarcoma [52]	Cell line: JJ012 Primary tumor -	Nanog	- Western blot	PRP-1	- PRP-1 decreased Nanog expression in JJ012 cells.
Synovial sarcoma [53]	Cell line: - Primary tumor 20 patients	CD133 CD29 CD44 Nestin ALDH1	- IHC	-	- CD133 was expressed in 85% of the samples. - ALDH was expressed in only 25% of the samples. ALDH+ cases were associated with a low survival rate. - CD29 and CD44 were expressed in 55% of the samples. - Nestin was expressed in 30% of the cases.
Undifferentiated cardiac sarcoma [54]	Cell line: - Primary tumor 5 patients	CD44 Oct ¾	- IHC	-	- CD44 marker was positive in all tumor samples. - Oct3/4 was not expressed in the nucleus in any sample.
GIST [55]	Cell line: GIST882 GIST48b Primary tumor 95 patients	CD133	- Tissue microarrays - IHC - PCR	5-aza-dC	- 5-aza-dC demethylation reactivated CD133 expression in cell lines. - The degree of CD133 methylation decreases with increasing tumor size. - Patients with higher CD133 expression had shorter survival. - CD133 was associated with gastric GIST, KIT mutation, and poor survival.
GIST [56]	Cell line: - Primary tumor 27 patients	CD133 CD90 CD44 CD34	- IHC - Flow cytometry	Imatinib	- Cells with mutated KIT from tumor samples were negative for CD45, positive for CD133, CD90, CD44, and CD34. - The c-kit Exon 11-mutated and treated samples showed lower expression of KIT, CD133, CD90, and increased CD34 compared to untreated samples.
Liposarcoma Osteosarcoma Synovial sarcoma Fibrosarcoma Leiomyosarcoma Rhabdomyosarcoma Schwannoma Alveolar sarcoma Clear cell sarcoma Chondrosarcoma [57]	Cell line: - Primary tumor: 38 STS 16 osteosarcoma 43 metastasis 7 local recurrence 4 primary tumor	ALDH CD133	- FACS (CD133+) - ALDH activity	Doxorubicin Ifosfamide Gemcitabine Docetaxel Cisplatin Etoposide	- There was higher expression of ALDH in STS than in osteosarcoma. Moreover, the expression was higher in clonogenic STS cultures. - No differences were found for CD133 markers.

Fibroblast growth factor (FGF), epidermal growth factor (EGF), magnetic-activated cell sorting (MACS), fluorescent-activated cell sorter (FACS), immunohistochemistry (IHC), 5-aza-2′-deoxycytidine (5-aza-dC), proline rich polypeptide-1 (PRP-1).
